# Seven years post-Fukushima: long-term measurement of exposure doses in Tomioka Town

**DOI:** 10.1093/jrr/rry082

**Published:** 2018-11-05

**Authors:** Akira Tsukazaki, Yasuyuki Taira, Makiko Orita, Noboru Takamura

**Affiliations:** Department of Global Health, Medicine and Welfare, Atomic Bomb Disease Institute, Nagasaki University, 1-12-4 Sakamoto, Nagasaki, Japan

*To the editor*:

Seven years have passed since the nuclear accident at the Fukushima Dai-ichi Nuclear Power Station (FDNPS). Following the accident, the prime minister issued an evacuation order [1, 2]. This order forced residents of Tomioka, located within 10–20 km of the FDNPS, to evacuate just after the accident. Post-accident, Tomioka continues recovery efforts in the town through repairing of infrastructure damaged by the earthquake and tsunami, and decontamination. In April 2013, 2 years after the accident, ambient doses in Tomioka were ranging from 0.75 to 4.10 μSv/h, whereas those in June 2018, 7 years after the accident, they were ranging from 0.08 to 3.00 μSv/h [3]. After allowing temporary visits and stays for the residents in preparation for their return, the Japanese government lifted the evacuation order for Tomioka on 1 April 2017, excluding the ‘difficult-to-return zone’. By March 2018, only 4.2% (561 of 13 172) of the residents had returned to Tomioka. Many factors may be associated with residents’ hesitation to return, such as insufficient recovery of infrastructure, commercial facilities and educational institutions, delayed reconstruction of residential houses, residents establishing their lives elsewhere (mostly in urban areas), and insufficient employment opportunities in Tomioka. But anxiety about radiation exposure remains one of the biggest reasons why residents hesitate to return to Tomioka.

In April 2017, we established a satellite office in Tomioka to support the recovery efforts, in cooperation with residents and the town office. In this satellite office, we have conducted risk assessment of residents of Tomioka, based on their individual exposure doses evaluated by dosimeters.

A 70-year-old man who returned to Tomioka visited our satellite office for risk assessment by our staff. He had been making temporary visits to Tomioka since April 2013 and returned permanently in April 2017 to clean up his house for his return, but he did not participate in the decontamination work. During his visits to Tomioka, he recorded the lengths of his visits and the ambient dose rates in his house using the ambient dosimeter (DOSEe®, Fuji Electric Corp., Ltd, Tokyo, Japan), and he carried a personal dosimeter (D-Shuttle®, Chiyoda Technol Corporation, Tokyo, Japan) to evaluate exposure doses.

**Fig. 1.**
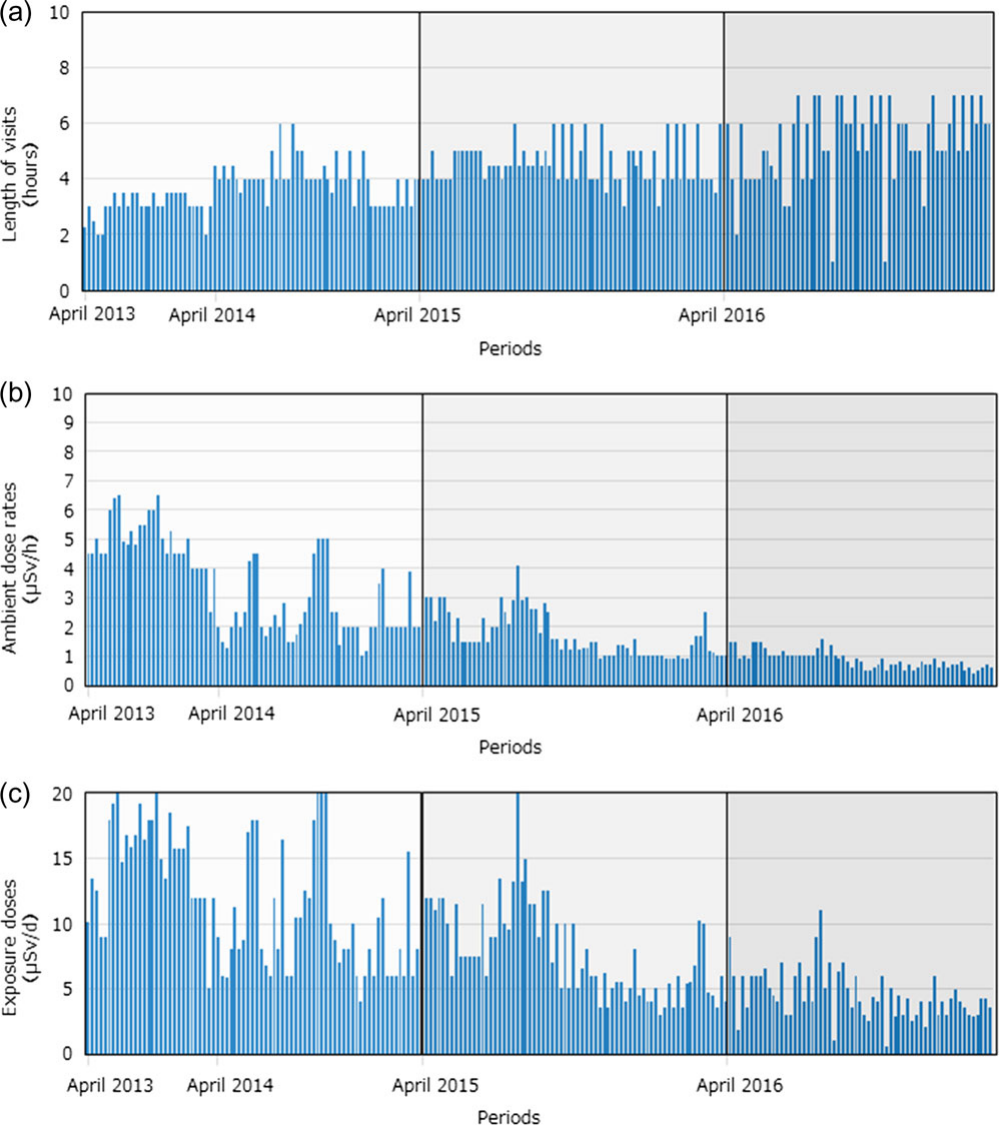
(a) Lengths of visits, (b) ambient dose rates, and (c) exposure doses in a resident of Tomioka between April 2013 and March 2015, April 2015 and March 2016, and April 2016 and December 2017.

The average length of his visits increased significantly in April–December 2016 (5.1 ± 1.4 h), compared with his visits in April 2013 – March 2015 (3.7 ± 0.8 h) and April 2015 – March 2016 (4.4 ± 0.8 h) (Fig. 1). His average daily exposure doses decreased significantly in April–December 2016 (4.7 ± 1.9 μSv/day) compared with those in April 2013 – March 2015 (12.5 ± 5.0 μSv/day) and in April 2015 – March 2016 (8.8 ± 3.7 μSv/day), parallel to the significant decrease in the ambient dose rate. His estimated annual external dose was 1.7 mSv in 2016.

His long-term measurements showed that, due to decontamination efforts and the natural decrease in radiocesium, external exposure doses dramatically decreased in his daily environment in Tomioka. Evaluation of personal dose measurements are important for risk assessment, because public ambient doses measurements conducted by a town office are undertaken by car-borne survey or by walking survey [3]. In addition to improving infrastructure, careful risk assessment (based on objective risk evaluation), of residents who have already returned or wish to return to Tomioka is essential. The role of specialists in this area is still very important for the recovery of Fukushima.
