# Corrigendum

**DOI:** 10.1093/jrr/rrz015

**Published:** 2019-05-24

Corrigendum to: Tsukasaki A, Taira Y, Orita M, et al. Seven years post-Fukushima: long-term measurement of exposure doses in Tomioka Town. *J Radiat Res* 2019; 60:159–160. https://doi.org/10.1093/jrr/rry082.

The author name Akira Tsukazaki was incorrect. It should have been Akira Tsukasaki. This has been corrected online.

